# Identification of resistance to cobweb disease caused by *Cladobotryum mycophilum* in wild and cultivated strains of *Agaricus bisporus* and screening for bioactive botanicals†

**DOI:** 10.1039/c9ra00632j

**Published:** 2019-05-14

**Authors:** Idrees Muhammad, Frederick Leo Sossah, Yang Yang, Dan Li, Shoujian Li, Yongping Fu, Yu Li

**Affiliations:** Engineering Research Center of Chinese Ministry of Education for Edible and Medicinal Fungi, Jilin Agricultural University Changchun 130118 P. R. China yongpingfu81@126.com yuli966@126.com +86-431-8453-2989

## Abstract

Outbreaks of cobweb disease are becoming increasingly prevalent globally, severely affecting the quality and yield of *Agaricus bisporus*. However, cobweb disease-resistant strains are rare, and little is known regarding the biocontrol management of the disease. Here, we isolated a pathogen from a severe outbreak of cobweb disease on *A. bisporus* in China and identified it as *Cladobotryum mycophilum* based on morphological characteristics, rDNA sequences, and pathogenicity tests. We then tested 30 *A. bisporus* strains for cobweb disease resistance by inoculating with *C. mycophilum* and evaluated the activity of different botanicals. We found that two wild strains of *A. bisporus* originating from the Tibetan Plateau in China were resistant to cobweb disease, and four commercial strains were susceptible. Yield comparisons of the inoculated and uninoculated strains of *A. bisporus* with *C. mycophilum* revealed yield losses of 6–38%. We found that seven botanicals could inhibit *C. mycophilum* growth *in vitro*, particularly *Syzygium aromaticum*, which exhibited the maximum inhibition (99.48%) and could thus be used for the further biocontrol of cobweb disease. Finally, we identified the bioactive chemical constituents present in *S. aromaticum* that could potentially be used as a treatment for *C. mycophilum* infection using Fourier transform infrared (FTIR) spectroscopy. These findings provide new germplasm resources for enhancing *A. bisporus* breeding and for the identification of botanicals for the biocontrol of cobweb disease.

## Abstract 2

Outbreaks of cobweb disease are becoming increasingly prevalent globally, severely affecting the quality and yield of *Agaricus bisporus*.

## Graphical abstract

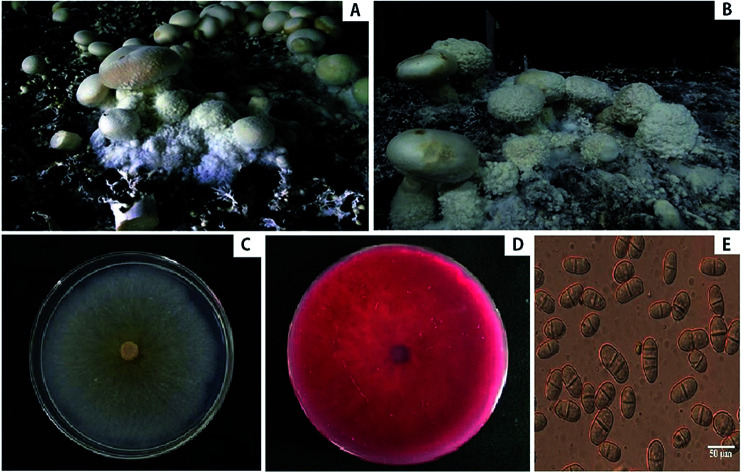

## Introduction

Cobweb disease, caused by *Cladobotryum*, is considered one of the most devastating fungal diseases impacting mushroom cultivation worldwide, resulting in significant yield and quality losses.^1,2^ In the early 1990s, yield losses of up to 40% were reported in *Agaricus bisprous* due to the frequent epidemics of cobweb disease in Ireland and Britain.^3^ Cobweb disease has been extensively reported and is caused by various fungal species, including *C. mycophilum*, *C. varium*, *C*. *dendroides*, and *C. protrusum*.^3,4^ Among these, *C. mycophilum* can cause severe cobweb symptoms on cultivated mushrooms, including *Pleurotus eryngii*, *A. bisporus*, and *Ganoderma lingzhi*.^5–9^

*Agaricus bisporus*, commonly known as button mushroom, is a widely cultivated edible mushroom that is high in protein and low in fat. With the expansion of the commercial cultivation of these mushrooms, the occurrence of fungal diseases on *A. bisporus* has also increased. In 2016, cobweb disease with an incidence of 2–5% on a commercial *A. bisporus* farm in China was first identified as being caused by *C. mycophilum*.^10^ Though the resistance of *A. bisporus* strains to different pathogens has been studied,^11–13^ an evaluation of cobweb disease resistance/susceptibility to *Cladobotryum* species has neither been detected nor well characterized.

Fungicide application is not suitable for palatable mushrooms owing to their residual toxicity.^14^ Furthermore, some species of *Cladobotryum* are resistant to fungicides.^15^ Previous studies have shown that certain plant extracts exhibit antimicrobial activity and have been used traditionally for the treatment of plant and animal diseases,^16–19^ such as *Mentha longifolia*, *Allium sativum*, and *Syzygium aromaticum*. The application of these substances of natural origin as mushroom crop protectants could constitute a convenient, low-cost, and safe solution for both humans and the environment and is also compatible with the natural ecosystem.

Therefore, to accelerate the breeding of *A. bisporus* strains with resistance to *C. mycophilum* and implement disease management approaches for the cultivation of *A. bisporus*, we designed our study with the following objectives: (1) to screen cultivated and wild strains of *A. bisporus* in order to identify resistance to *C. mycophilum*; and (2) to evaluate different botanicals for *in vitro* antifungal activity towards the cobweb disease pathogen *C. mycophilum* and assess the molecular spectra of the most effective botanical by Fourier-transform infrared spectroscopy (FTIR).

## Materials and methods

### Collection, isolation, and identification of the pathogen causing cobweb disease on *A. bisporus*

Fruiting bodies of *A. bisporus* showing typical symptoms of cobweb disease were collected from a commercial mushroom cultivation factory in Chengde, Hebei province, China. The pathogen was isolated from diseased fruiting bodies of *A. bisporus* and cultured on potato dextrose agar (PDA) medium. The pathogen was then identified to the species level based on its morphological, culture, and molecular characteristics.^4,6^ Genomic DNA was extracted from the *C. mycophilum* cultures according to the manufacturer protocol (KANGWEI, Beijing, China). PCR amplification was performed for the internal transcribed spacer (ITS) gene region using universal set of primers ITS1 and ITS4,^20^ and the translation elongation factor (TEF) 1-alpha gene using EF-1 and EF-2 primers.^21,22^ The PCR system comprised of an initial step of DNA denaturation at 94 °C/4 min, proceeded by 28 cycles involving 94 °C/50 s, 50 °C/50 s (ITS) or 52 °C/50 s (TEF) and 72 °C/1 min with a final extension at 72 °C/10 min. The expected band size was selected, purified and subjected to sequence analysis at Sangon Biotech Co., Ltd. (Shanghai, China). These sequencing results were compared to the sequence of *Cladobotryum* in the GenBank of NCBI. MEGA-7 software^23^ was then used to construct the phylogenetic tree using Neighbor-joining (NJ) method.^24^

The pathogenicity of *C. mycophilum* isolate CMIDR1 was tested using Koch's postulates, conducted on the commercial *A. bisporus* strain CCMJ1009. CCMJ1009 was cultivated in a controlled incubation and fruiting room at the Edible Mushroom Base of Jilin Agricultural University, China. The cultivation of *A. bisporus* CCMJ1009 was performed as described in Fu *et al.* (2016). In the first set of experiments, the spore suspension of *C. mycophilum* CMIDR1 (7.5 × 10^3^ spores per mL) was prepared on the day of inoculation from a 5 day-old culture on PDA, and then the concentration of the spore suspension was determined by means of a hemocytometer. Healthy fruiting bodies of CCMJ1009 were then inoculated with 50 μL of prepared spore suspension of *C. mycophilum* maintained at a temperature of 22 °C and 95% humidity.^10^ Sterilized distilled water (SDW) was used for the control group. In the second set of experiments, a spore suspension of *C. mycophilum* CMIDR1 (7.5 × 10^3^ spores per mL) was prepared on the 9^th^ day of casing and sprayed onto the surface of the casing layer (20 mL per basket, 35 × 25 × 17 cm), while the control baskets were sprayed with SDW.^25^ The casing surface was checked daily to examine the isolated outbreaks. The affected area and infected fruiting bodies were treated by covering with damp paper and salt to prevent the dry conidia from being released and to avoid the possibility of cross contamination.^6^ Then, the pathogen strains from the diseased button and caps were re-isolated and cultured on PDA. Genomic DNA of the pathogen was extracted, and PCR amplification and sequencing of ITS gene was performed. The methods were same as the above mentioned. The sequencing results were compared to the sequence of the CMIDR1 strain.

### Evaluation of *A. bisporus* strains for cobweb disease resistance caused by *C. mycophilum*

A total of 30 commercial and wild *A. bisporus* strains were evaluated for resistance/susceptibility to cobweb disease caused by *C. mycophilum* (Table 1). The 15 wild strains originated from three provinces in China *viz.* Sichuan, Shanxi, and Yunnan. The 15 commercial strains of *A. bisporus* were collected from different countries. All the strains of *A. bisporus* used in this study were cultured on PDA media at 25 °C for two to three weeks and preserved at 4 °C for further use, at the Engineering Research Centre of the Chinese Ministry of Education for Edible and Medicinal Fungi, Jilin Agricultural University, Changchun, China.

**Table 1:** Strains of *A. bisporus* used in this study for cobweb disease resistance

Strain name	Original reference	Origin	Strain types
CCMJ1009	A15	USA	Cultivated
CCMJ1013	As2796	China	Cultivated
CCMJ1020	ZA	Germany	Cultivated
CCMJ1021	S130A	USA	Cultivated
CCMJ1018	As4580	China	Cultivated
CCMJ1028	S46	China	Cultivated
CCMJ1033	C13	USA	Cultivated
CCMJ1035	0072	USA	Cultivated
CCMJ1037	U1	Netherlands	Cultivated
CCMJ1038	PSU310	USA	Cultivated
CCMJ1039	126	Netherlands	Cultivated
CCMJ1053	M-1	Spain	Cultivated
CCMJ1109	Ag23	England	Cultivated
CCMJ1343	W192	China	Cultivated
CCMJ1352	A12	USA	Cultivated
CCMJ1106	2094	Tibet, China	Wild
CCMJ1347	T12387	Yunnan, China	Wild
CCMJ1351	W2	Sichuan, China	Wild
CCMJ1350	W1	Sichuan, China	Wild
CCMJ1360	W3	Sichuan, China	Wild
CCMJ1361	W4	Sichuan, China	Wild
CCMJ1363	W5	Sichuan, China	Wild
CCMJ1369	W6	Sichuan, China	Wild
CCMJ1372	W7	Sichuan, China	Wild
CCMJ1374	W11	Sichuan, China	Wild
CCMJ1377	W8	Sichuan, China	Wild
CCMJ1384	W10	Sichuan, China	Wild
CCMJ1110	W13	Shanxi, China	Wild
CCMJ1379	W12	Sichuan, China	Wild
CCMJ1381	W9	Sichuan China	Wild

Screening and yield loss assessment of the resistance of *A. bisporus* strains against cobweb disease were performed on March 2017, August 2017, and April 2018. At each assessment, all 30 mushroom strains were cultivated with the same batch of compost and casing soil in twin rooms. The spore suspension was prepared from *C. mycophilum* CMIDR1, and the inoculation method was the same as mentioned above. For each *A. bisporus* strain, nine trays were dripped with the spore suspension of *C. mycophilum*, and another nine trays were dripped with sterile distilled water (20 mL) as a control treatment. After 15–20 days of casing, when the *A. bisporus* mycelia had fully permeated the casing layer, the room temperature was dropped down to 16–18 °C for fruiting purposes.

The level of resistance of the mushroom strains to cobweb disease caused by *C. mycophilum* was classified on the basis of visual assessment during the first three flushes by using a modified disease rating scale detailed in Back *et al.* (2012),^15^ as follows: 0 = no visible disease development (immune, I); 1 = 1–10% disease severity (resistant, R); 2 = 11–30% disease severity (moderately susceptible, MS); and 3= >30 disease severity (susceptible, S).

### Screening of botanicals for antifungal activity and FTIR spectra

The aqueous extracts of the seven botanicals were evaluated *in vitro* using the poisoned food technique^26^ on PDA against both *A. bisporus* and *C. mycophilum* at five different concentrations of 1%, 2%, 3%, 4%, and 5% of aqueous extracts (10% w/v), including mint leaves and stem (*Mentha longifolia*), garlic bulb (*Allium sativum*), turmeric rhizome (*Curcuma longa*), ginger rhizome (*Zingiber officinale*), clove seeds/buds (*Syzygium aromaticum*), cinnamon seeds (*Cinnamomum zeylanicum*), and neem leaves (*Azadirachta indica*). The dried botanicals were purchased from the local market, cleaned, and crushed into a fine powder using an electric grinder. Stock aqueous extracts were first prepared by soaking 100 g of powdered plant material in 1000 mL sterilized distilled water (10% w/v) at room temperature for 24 h with occasional shaking. The mixture was then strained through two layers of sterilized muslin cloth followed by Whatman No. 1 filter paper under aseptic conditions and stored at 4 °C (ref. 27) and were generally used within one week to avoid any potential chemical alterations.^26^ The test concentrations of the botanicals were then aseptically amended with molten PDA (50 °C) medium and poured into Petri plates. Five replications were maintained for each concentration and for the control. Five-milliliter discs of *C. mycophilum* (4 days old) and *A. bisporus* (15 days old) were placed in the center of the Petri plates. The comparative efficacy of the aqueous extracts was calculated as the percentage mycelial growth inhibition of the test fungus in each treatment using the following formula:^18^ mycelial inhibition (%) = (*C* − *T*)/*C* × 100, where *C* = radial mycelial growth (mm) of the control and *T* = radial mycelial growth of the treatment (mm).

The molecular spectra of the most effective botanicals were obtained by FTIR (Bruker, Vertex-70). The ground samples were mixed with potassium bromide (KBr) powder and pressed into tablet-shaped pellets under pressure, and the spectra were recorded at a frequency range of 400–4000 cm^−1^.^28^

### Data analysis

Harvesting of the *A. bisporus* fruiting bodies was carried out for three successive flushes. The yields of the fruiting bodies were determined on the basis of the harvested 2^nd^ stage fruiting bodies per basket. The fruiting bodies were harvested daily for all the strains from both the twin rooms, and the number and weight were recorded for each strain. To compare the control and inoculated treatments, we applied *t*-statistics at a significance level of *α* = 0.05 and *α* = 0.01. The results were reported on the basis of the mean values from three cultivation cycles.

## Results

### Identification of the pathogen responsible for cobweb disease on *A. bisporus*

**Fig. 1:**
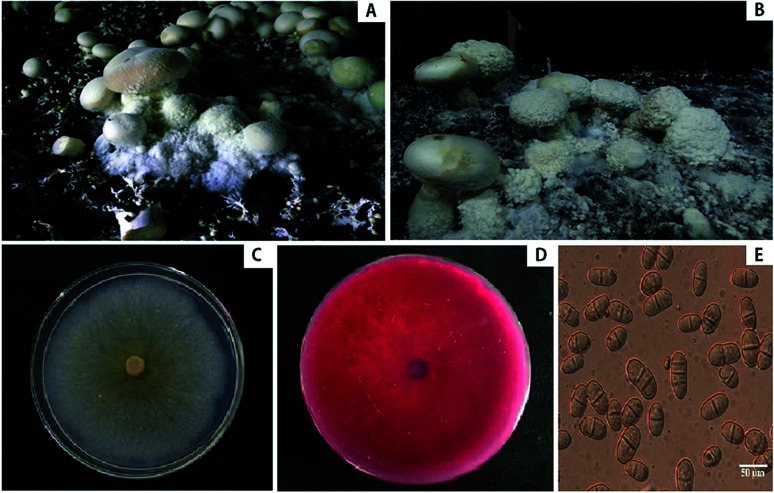
The cobweb disease of *A. bisporus* caused by *C. mycophilum*. (A and B) Natural infection of cobweb disease in mushroom cultivation factory in Hebei, China. Cobweb symptoms on diseased fruiting bodies of *A. bisporus*: fluffy mycelium over the casing soil and the dense mass of sporulation engulfing the fruit bodies. (C and D) Cultural characteristics of isolate of *C. mycophilum* on PDA from *A. bisporus* farms ((C) front side after 3 days; (D) back side after 25 days). (E) Morphological characters of isolates, conidia with 1–3 septa.

During the fall of 2016, which is the production season of *A. bisporus*, we noticed cobweb disease symptoms on *A. bisporus* in Chengde, Hebei province, China. The symptoms initially appeared as the development of whitish grey mycelia on the fruiting bodies of *A. bisporus* and the casing soil (Fig. 1A). As the disease progressed, the effuse mycelia of the pathogenic fungi grew rapidly and gradually expanded to cover the host stipes, caps, and primordia (Fig. 1B). Eventually, the entire fruiting bodies began to rot (Fig. 1B) and the disease incidence ranged from 5% to 8%. We isolated the pathogen associated with cobweb disease on *A. bisporus* and identified it based on morphological characteristics, ITS and tef1 rDNA sequences, and pathogenicity tests.

The isolates of *C. mycophilum* grew radially and covered the entire Petri plates within 3–4 days. The colonies appeared white in the early stages (Fig. 1C), slowly producing yellow pigments within 5 days, and the entire medium turned red about 10–14 days later (Fig. 1D). The growth rate of these isolates reached 23 mm day^−1^ on PDA at 25 °C. Chlamydospores were more easily observed on the older mycelia (Fig. 1E). The conidia (*n* = 50) were transparent, single-celled, oval to oblong with diaphragms, possessed 1–3 septa, and ranged 5.9–13.1 × 9.9–31.0 μm in size. Based on the morphological characteristics, the pathogen was identified as *C. mycophilum*.

**Fig. 2:**
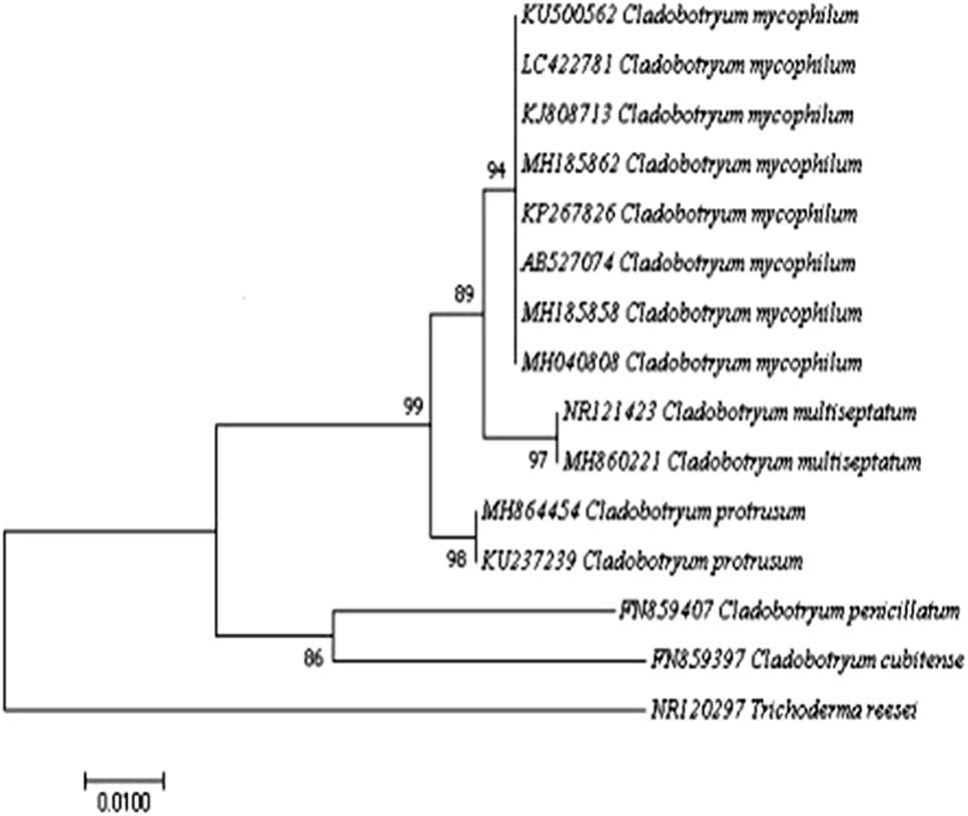
The phylogenetic tree constructed by the neighbor-joining method based on comparison of the internal transcribed spacer (ITS) gene. Sequences of *Cladobotryum mycophilum* LC422781 with those of other *Cladobotryum* species from GenBank. *Trichoderma reesei* was used as the out-group. The bootstrap test (1000 replicates) is shown next to the branches. The tree is drawn to scale, with branch lengths in the same units as those of the evolutionary distances used to infer the phylogenetic tree.

The representative isolate CMIDR1 of *C. mycophilum* was used for further molecular identification. The generated ITS and tef1 sequences were searched with BLASTn and were found to share 99% similarity with published *C. mycophilum* sequences in GenBank. The phylogenetic tree based on ITS included other published *Cladobotryum* sequences (Fig. 2) and showed that CMIDR1(LC422781) clustered with other *C. mycophilum* accessions and was closely related to *C. multiseptatum*. Thus, based on the morphological and molecular characteristics, we confirmed that the fungus was *C. mycophilum*.

**Fig. 3:**
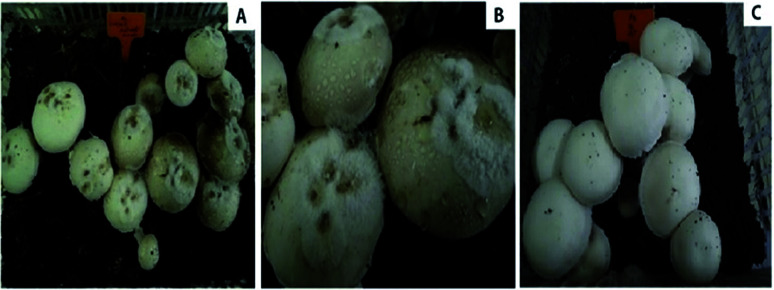
Pathogenicity test of *C. mycophilum* on *A. bisporus* strain CCMJ1009. (A and B) Symptoms of cobweb disease after 3 days inoculation; (C) control.

We then used Koch's postulates to confirm the pathogenicity of isolate CMIDR1 of *C. mycophilum* on *A. bisporus* (Fig. 3). The fruiting bodies developed visible cobweb symptoms on the 4–5^th^ day after inoculation of the CMIDR1 strain. White mycelia of CMIDR1 appeared and spread rapidly on the surfaces of the fruiting bodies of *A. bisporus*. Disease symptoms were also noticed on the casing surface and then on the primordial and fruiting bodies after 16 days of inoculation. These symptoms were consistent with the symptoms observed in the above-mentioned commercial mushroom cultivation company (Fig. 1A and B) in Hebei province, China. Thus, Koch's postulates confirmed that the pathogen re-isolated from the diseased fruiting bodies of *A. bisporus* was *C. mycophilum*.

### Identification of cobweb disease resistance and yield loss assessment

**Fig. 4:**
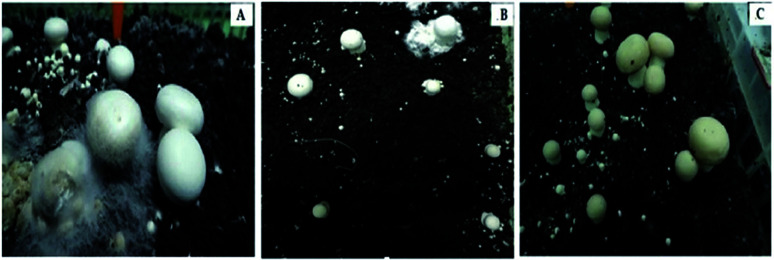
The resistance level of *A. bisporus* strains to cobweb disease. (A) Susceptible strain; (B) moderately susceptible strain; (C) resistant strain.

The mycelia of *A. bisporus* aggregated after 14–18 days of inoculation with the *C. mycophilum* CMIDR1 spore suspension. Based on the disease rating scale for cobweb disease assessment, four commercial strains of *A. bisporus* (Table 2), including CCMJ1009, CCMJ1013, CCMJ1035, and CCMJ1109, were found to be susceptible (S) to the cobweb disease caused by *C. mycophilum*. We also found 24 strains of *A. bisporus*, including CCMJ1018, CCMJ1020, CCMJ1028, CCMJ1031, CCMJ1033, CCMJ1037, CCMJ1038, CCMJ1039, CCMJ1106, CCMJ1110, CCMJ1343, CCMJ1347, CCMJ1350, CCMJ1352, CCMJ1053, CCMJ1360, CCMJ1361, CCMJ1363, CCMJ1369, CCMJ1372, CCMJ1374 CCMJ1377, CCMJ1379, and CCMJ1381, which were moderately susceptible (MS) to cobweb disease. Among these strains, 11 constitute cultivated strains and thirteen constitute wild strains. More than 10% of the primordia and 1^st^ and 2^nd^ stage fruiting bodies in these strains were severely damaged by the disease (Fig. 4). In contrast, in the control experiments lacking CMIDR1 spore suspension inoculation, the fruiting bodies of these 24 strains exhibited normal growth.

**Table 2:** Cobweb disease incidence and yield comparison of *A. bisporus* strains

Commercial strains	Yield (kg m^−2^)	Disease incidence (%)	Resistance level^a^	Wild strains	Yield (kg m^−2^)	Disease incidence (%)	Resistance level^a^
CCMJ1009	8.92 ± 0.375^a^	32.06 ± 4.642^ab^	S	CCMJ1110	8.22 ± 0.174^b^	17.67 ± 5.776^defghij^	MS
CCMJ1109	8.07 ± 0.264^abc^	37.84 ± 6.111^a^	S	CCMJ1350	6.09 ± 0.487^efgh^	15.75 ± 6.337^defghij^	MS
CCMJ1343	7.83 ± 0.706^abcd^	17.86 ± 3.990^defghij^	MS	CCMJ1384	6.08 ± 0.419^efgh^	8.28 ± 4.164^ij^	R
CCMJ1352	7.61 ± 0.160^bcd^	27.73 ± 3.361^abcd^	MS	CCMJ1351	6.04 ± 0.459^efgh^	6.49 ± 4.464^j^	R
CCMJ1020	7.56 ± 0.678^bcd^	19.52 ± 0.621^cdefghi^	MS	CCMJ1374	5.41 ± 0.902^ghijk^	13.08 ± 7.278^ghij^	MS
CCMJ1033	6.99 ± 0.809^cde^	16.00 ± 3.912^fghij^	MS	CCMJ1363	5.36 ± 0.681^ghijk^	10.84 ± 6.394^hij^	MS
CCMJ1035	6.96 ± 0.091^de^	30.55 ± 7.392^abc^	S	CCMJ1347	5.29 ± 0.262^ghijk^	24.88 ± 1.995^bcdefg^	MS
CCMJ1013	6.75 ± 0.667^def^	30.63 ± 6.143^abc^	S	CCMJ1361	5.13 ± 0.591^hijkl^	15.58 ± 4.472^efghij^	MS
CCMJ1039	6.35 ± 0.650^efg^	25.95 ± 5.703^abcdef^	MS	CCMJ1369	5.04 ± 0.232^hijkl^	25.04 ± 7.291^bcdefg^	MS
CCMJ1021	6.26 ± 0.796^efg^	27.46 ± 5.635^abcde^	MS	CCMJ1379	4.92 ± 0.314^ijkl^	20.63 ± 9.440^bcdefgh^	MS
CCMJ1053	5.80 ± 0.557^fghi^	25.48 ± 1.397^bcdef^	MS	CCMJ1377	4.72 ± 0.744^ijkl^	21.27 ± 7.860^bcdefgh^	MS
CCMJ1018	5.56 ± 0.926^ghij^	19.12 ± 5.679^cdefghi^	MS	CCMJ1372	4.71 ± 0.188^ijkl^	18.61 ± 8.935^cdefghi^	MS
CCMJ1038	5.42 ± 0.540^ghijk^	22.40 ± 2.510^bcdefgh^	MS	CCMJ1360	4.61 ± 0.835^jkl^	19.62 ± 3.799^cdefghi^	MS
CCMJ1028	5.39 ± 0.342^ghijk^	21.33 ± 6.398^bcdefgh^	MS	CCMJ1106	4.46 ± 0.233^kl^	12.36 ± 8.757^hij^	MS
CCMJ1037	5.35 ± 0.487^ghijk^	26.22 ± 4.306^abcdef^	MS	CCMJ1381	4.16 ± 0.337^l^	19.64 ± 11.674^cdefghi^	MS

a 0 = No disease development (immune, I); 1–10% disease severity (resistant, R); 11–30% disease severity (moderately susceptible, MS); >30% disease severity (susceptible, S). Values in the same column with the same following letters do not significantly differ (*p* < 0.05); ±standard deviation.

The two resistant (R) strains CCMJ1351 and CCMJ1384 formed primordia and fruiting bodies that exhibited only small damage, while normal growth was observed in the control treatments (un-inoculated). These two resistant strains were wild collections from southeast of Tibet in China. These findings demonstrated the importance of the wild germplasm from Tibet as sources of resistance to cobweb disease.

**Fig. 5:**
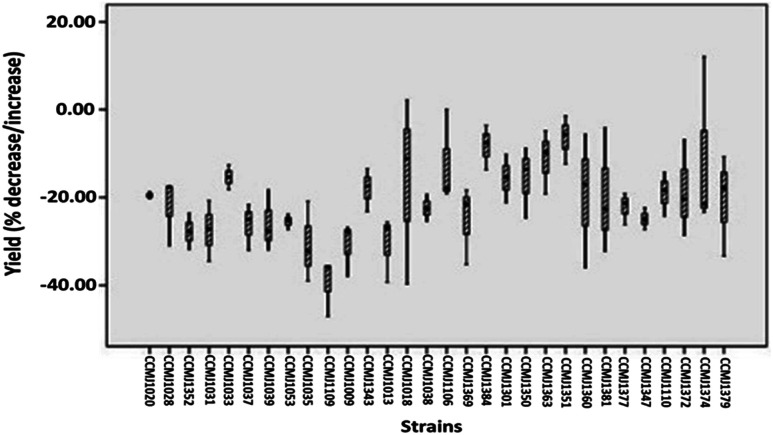
Percent decrease in total yield of 30 wild and commercial strains of *A. bisporus* inoculated with the cobweb disease pathogen *C. mycophilum* compared with the yield of uninoculated (control) strains by Box plot. Graph shows yield loss in all the strains of *A. bisporus* at different levels.

In terms of the commercial *A. bisporus* strains, CCMJ1009, CCMJ1018, CCMJ1035, CCMJ1038, CCMJ1109, and CCMJ1352 demonstrated highly significant (*P* < 0.01) yield loss, and the three strains CCMJ1028, CCMJ1037, and CCMJ1053 showed significant (*P* < 0.05) yield loss, while the rest of the commercial strains exhibited statistically non-significant (*P* > 0.05) yield loss (Table S1†). Similarly, of the wild *A. bisporus* strains, CCMJ1106, CCMJ1110, and CCMJ1347 demonstrated highly significant (*P* < 0.01) yield loss, and CCMJ1369, CCMJ1372, and CCMJ1381 exhibited significant (*P* < 0.05) yield loss, while the remainder of the wild strains displayed statistically non-significant (*P* > 0.05) yield loss (Table S1†). A commercial *A. bisporus* strain CCMJ1109 consistently demonstrated maximum yield loss (37.96%), followed by CCMJ1009 (31.06%) and CCMJA1035 (30.76%), while a wild strain CCMJ1351 (6.81%) showed (Fig. 5) the lowest yield loss followed by CCMJ1384 (8.52%) and CCMJ1363 (11.81%).

### Inhibition of the cobweb pathogen by the botanicals and FTIR spectra

**Fig. 6:**
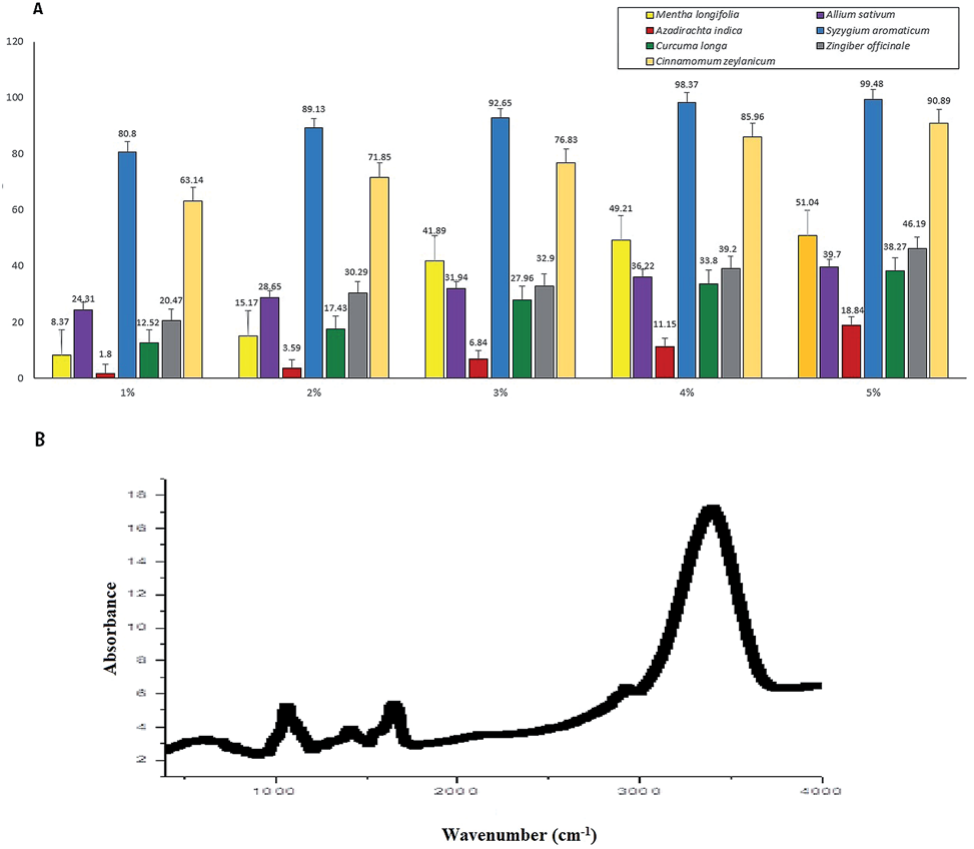
Inhibition of the cobweb pathogen by the botanicals and FTIR spectra. (A) Screening of botanicals at different concentrations, for *in vitro* inhibition study of *C. mycophilum*. (B) FTIR spectra of most toxic botanical *Syzygium aromaticum*.

We used aqueous extracts of the seven botanicals to evaluate the *in vitro* inhibition of the cobweb disease pathogen *C. mycophilum* (Fig. 6A). We found that *S. aromaticum* (99.48% inhibition over control) was most inhibitory, followed by *C. zeylanicum* (90.88%), *M. longifolia* (51.04%), *Z. officinale* (46.19%), *A. sativum* (39.70%), and *C. longa* (38.26%), while *A. indica* showed minimum inhibition (18.83%). Additionally, the tested botanicals were comparatively more effective at a concentration of five percent. These results indicated that the aqueous extracts of all the tested botanicals have antifungal properties, and marked variability was observed for the *in vitro* sensitivity of *C. mycophilum*.

For further insight into the structural properties of the botanicals, the FTIR spectrum of *S. aromaticum* was evaluated to identify the functional groups of the active components based on the peak value in the region of the infrared radiation. The FTIR analysis showed (Fig. 6B) a clear and maximum intensive peak at 3375 cm^−1^ that represents the OH groups; a moderate peak at 2852–2920 cm^−1^ that represents the frequency patterns of CH_2_ and CH_3_ groups; and a peak at 1730 cm^−1^ that represents an ester group C–O or aromatic ketone group C=O. Another peak at 1643 cm^−1^ represents the aromatic carbonyl group belonging to quinine.^29^ The other frequency peaks at 1452 and 1402 cm^−1^ indicated the presence of a CH_2_ group, and a peak at 1070 cm^−1^ represented the C–O group. Moderate peaks at 779 and 919 cm^−1^ showed the frequency patterns of the CH_2_ and C=C groups, respectively.^30^ The most prominent spectral bands as mentioned above mainly corresponding to the respective major antifungal compounds. Thus, the FTIR analysis identified novel phytochemical markers that could constitute useful analytical tools for assessing the quality of the sample and identifying the presence of functional groups in *S. aromaticum*. We could further isolate and screen for different biological activities depending on their antifungal uses.

## Discussion

*Cladobotryum mycophilum* has been identified as the causal agent of cobweb disease on cultivated mushrooms in Europe, Africa, and Asia.^31^ Based on morphological and molecular characteristics and Koch's postulates, we confirmed that the myco-pathogenic fungus *C. mycophilum* was the causal agent of cobweb disease on *A. bisporus*. The pathogen produced fluffy white mycelia on the casing layer, primordia, and fruiting bodies, followed by cap spotting and decay of the infected mushrooms, eventually resulting in unmarketable mushrooms. The cobweb disease symptoms were similar to those reported by previous studies.^2,5,6,8,32^ In China, *C. mycophilum* has been reported to cause cobweb disease on *A. bisporus* and *G. lingzhi*.^9,10^ In addition, a cross-pathogenicity study found that *Flammulina velutipes* might be a potential host for *C. mycophilum*.^15^ In the future, an effective monitoring program and appropriate disease control methods are required to prevent economic losses. *Cladobotryum mycophilum* produces an abundance of spores that are easily dispersed by the air in factories, which could lead to the rapid development of cobweb disease epidemics.^15^ Thus, preventive and control measures could mainly focus on eliminating the route of pathogen transmission through enhanced hygiene and protection against secondary infection.^8,31^ The sensitivity of commercial fungicides on *C. mycophilum* and their selectivity on the host require further examination, which could help regular resistance monitoring against fungicides^33^ and *A. bisporus* disease management strategies should be focused on integrated disease control programs.^34^

The wild strains of *A. bisporus* contained more genetic diversity than the commercial cultivars.^12^ However, no previous reports regarding the resistance/susceptibility and yield losses of cobweb disease on wild strains exist. Our results indicated clear differences in yield losses between the *A. bisporus* strains (Fig. 4). To obtain a broader analysis of *A. bisporus* resistance to cobweb disease, we used cultivated strains and new germplasm resources from the wild strains that occur naturally in the harsh environment. This is the first report regarding the evaluation of the resistance of wild and commercial *A. bisporus* strains to cobweb disease. Previous studies showed that the wild resources of *A. bisporus* in the Tibetan Plateau of China have a high degree of variation in resistance to wet bubble disease (WBD), and 10 wild strains were highly resistant to WBD caused by *Hypomyces perniciosus* (formerly *Mycogone perniciosa*).^12^ However, we only found two strains that were resistant to cobweb disease caused by *C. mycophilum*. Of these two strains, we previously found CCMJ1351 to be highly resistant to WBD. We suggest that CCMJ1351 can be used for the breeding of highly disease-resistant cultivars.

Cobweb disease was observed in every flush, but maximum crop damage was observed during the third flush irrespective of the commercial or wild strain, as the disease incidence increased with crop age.^6^ However, there was a late appearance of the disease in the wild strains in comparison to the commercial strains, and two wild strains from China exhibited resistance against the cobweb disease pathogen. The results of this study provide useful information regarding the response of commercial and wild germplasm resources of *A. bisporus* to cobweb disease as well as potential wild germplasm resources for disease resistance.

According to previous reports, yield losses caused by cobweb disease in commercial *A. bisporus* strains vary from 10–29% to 22–62%.^35,36^ The environmental conditions during the experimental period and the strains of cobweb pathogen used might be responsible for these differences. It should be emphasized that there were insufficient data and studies available on the resistant and susceptible strains of *A. bisporus* towards *Cladobotryum*. The current investigation elucidated the resistant and susceptible wild and commercial *A. bisporus* strains that might have implications for future breeding programs and the selection of strains for the commercial cultivation of button mushroom and for the management of cobweb disease outbreaks.

The identification of new fungicides that are effective and biodegradable and exhibit increased selectivity is necessary for reducing the use of phytotoxic chemicals. Natural plant-derived products are comparatively safer and could be integrated into disease management programs as they possess antifungal activity without being phytotoxic.^37^ In this study, clove was found to be highly effective at inhibiting the growth of *C. mycophilum*. Earlier studies discovered that clove extract was highly active against many fungal genera, such as *Aspergillus*, *Cladosporium*, *Penicillium*, *Rhizopus*, and *Saccharomyces*, which is in accordance with our study.^38,39^ Our results corroborate those of previous studies where clove and cinnamon essential oils were able to suppress mycopathogens, with clove oil exhibiting greater toxicity than cinnamon to *Lecanicillium fungicola* and *Cladobotryum dendroides*.^40^

The bio-efficacy of botanicals is attributed to the fact that they have active compounds, such as azadirachtin, allicin, and salicin, which exhibit antifungal, antibacterial, and anti-insecticidal properties in nature.^41^ Some research has also reported that botanical extracts and oils used in different concentrations can effectively control the mycelial growth of mycopathogens.^42–44^ Clove oil contains eugenol up to 70–85% which is a phenolic compound with high antimicrobial activity.^40^ The antifungal activity of the botanicals in the present study might be due to the presence of one or more bioactive compounds, such as alkaloids, glycosides, flavonoids, steroids, and saponins.^45^ The maximum intensive peak observed at 3375 cm^−1^ represented the OH groups; a peak at 1730 cm^−1^ and 1070 cm^−1^ represented the group C–O that is the characteristic band for alcohols and phenols; and a peak at 1643 cm^−1^ represented the aromatic carbonyl group belonging to quinine. Therefore, clove contains various bioactive components with a high degree of antifungal activity against the cobweb disease pathogen. Our study provides a foundation for the use of some compounds as new and more potent natural antifungal products, as FTIR spectroscopy has been proved to be a reliable and sensitive method for the detection of biomolecular composition.^46^ Further *in vivo* study is in progress for screening of botanicals for cobweb disease management and detailed identification and application of bioactive compounds.

## Conclusion

We discovered that the severe outbreak of cobweb disease on *A. bisporus* in China was caused by *C. mycophilum*, which is known to be the fungal pathogen associated with cobweb disease globally. All of the wild and cultivated *A. bisporus* strains showed different responses to cobweb disease resistance. Almost all of the cultivated strains of *A. bisporus* exhibited different levels of susceptibility, while the two wild strains from the Tibetan Plateau demonstrated potential resistance to cobweb disease caused by *C. mycophilum*. This is the first evaluation of the resistance of wild and commercial *A. bisporus* strains to cobweb disease. Additionally, the results of this study provided insight into the use of botanicals for the fungicide-free cultivation of mushrooms. Further studies regarding the use of these resistant wild germplasm resources in future breeding programs for stable resistance and genetic studies of *A. bisporus* strains are required. Furthermore, greater insight into the host–pathogen interaction is required for the reduction of crop losses in the future.

## Conflicts of interest

There are no conflicts to declare.

## Supplementary Material

RA-009-C9RA00632J-s001
