# Supplementary material for: Identification of resistance to cobweb disease caused by Cladobotryum mycophilum in wild and cultivated strains of Agaricus bisporus and screening for bioactive botanicals
Source: RSC Adv. 2019 May 14;9(26):14758–65. doi: 10.1039/c9ra00632j (PMC9064161; doi:10.1039/c9ra00632j)

Electronic Supplementary Material (ESI) for RSC Advances.
This journal is © The Royal Society of Chemistry 2019

Table S1. Comparison between the Control and Inoculated (*C. mycophilum*) treatments regarding yield loss for Wild and Commercial strains of *A. bisporus*.

Commercial strain	Type	Mean	t-value	Wild Strains	Type	Mean	t-value
CCMJ1020	Control	661.89±72.61	2.31	CCMJ1106	Control	390.23±24.97	1.58**
	Inoculated	533.00±63.53			Inoculated	341.95±46.52	
CCMJ1028	Control	471.92±36.67	3.25*	CCMJ1369	Control	440.99±24.87	4.13*
	Inoculated	367.50±41.88			Inoculated	330.18±39.24	
CCMJ1352	Control	665.65±17.17	9.67**	CCMJ1384	Control	532.76±44.99	1.54
	Inoculated	481.00±28.25			Inoculated	487.33±24.31	
CCMJ1021	Control	547.98±85.32	2.53	CCMJ1361	Control	449.13±63.38	1.47
	Inoculated	396.08±59.64			Inoculated	378.65±53.94	
CCMJ1033	Control	611.82±69.07	1.76	CCMJ1350	Control	532.82±52.15	1.86
	Inoculated	517.74±61.67			Inoculated	448.88±58.39	
CCMJ1037	Control	467.93±52.18	3.99*	CCMJ1363	Control	469.30±58.39	1.09
	Inoculated	343.45±14.13			Inoculated	413.83±48.64	
CCMJ1039	Control	555.67±69.69	2.12	CCMJ1351	Control	528.26±49.24	1.18
	Inoculated	414.57±91.96			Inoculated	492.25±19.36	
CCMJ1053	Control	508.18±59.78	2.79*	CCMJ1360	Control	403.46±29.50	1.65
	Inoculated	379.38±52.99			Inoculated	325.83±75.97	
CCMJ1035	Control	609.27±20.20	5.72**	CCMJ1381	Control	364.19±36.17	3.06*
	Inoculated	421.83±52.99			Inoculated	289.24±22.19	
CCMJ1109	Control	706.83±25.64	7.74**	CCMJ1377	Control	412.70±52.15	2.62
	Inoculated	428.49±56.75			Inoculated	320.04±31.94	
CCMJ1009	Control	780.34±40.25	8.80**	CCMJ1347	Control	462.50±28.11	4.73**
	Inoculated	537.93±25.64			Inoculated	347.76±31.21	
CCMJ1343	Control	685.00±75.69	2.40	CCMJ1110	Control	719.06±31.43	7.14**
	Inoculated	560.17±48.63			Inoculated	581.46±11.17	
CCMJ1013	Control	590.58±71.52	2.73	CCMJ1372	Control	412.64±20.21	3.48*
	Inoculated	412.61±87.27			Inoculated	334.64±33.15	
CCMJ1018	Control	486.93±99.29	1.50**	CCMJ1374	Control	473.94±96.63	1.12
	Inoculated	394.15±39.51			Inoculated	409.80±22.27	
CCMJ1038	Control	474.34±57.96	2.23**	CCMJ1379	Control	430.40±33.70	1.99
	Inoculated	369.15±57.73			Inoculated	343.25±67.81	

Non-significant (P>0.05); * = Significant (P<0.05); ** = Highly significant (P<0.01)

Fig. S2: Screening of botanicals

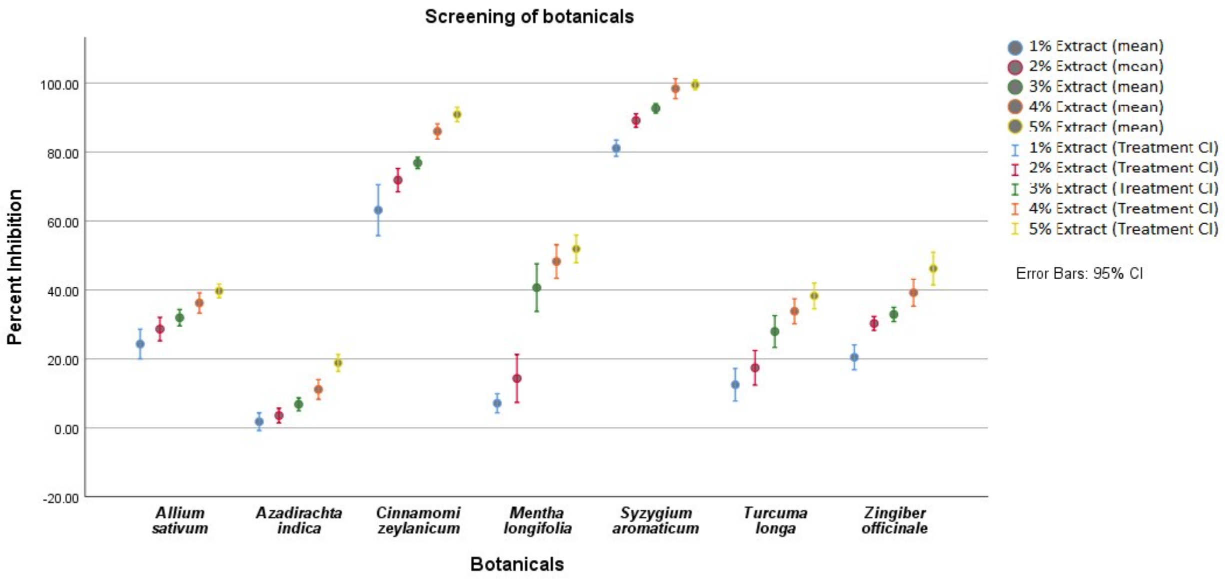

Supplement: RA-009-C9RA00632J-s001 [file RA-009-C9RA00632J-s001.pdf]
